# Supplementary material for: Sub-THz Vibrational Dynamics in Ordered Mesoporous Silica Nanoparticles
Source: Nanomaterials (Basel). 2023 Jul 15;13(14):2078. doi: 10.3390/nano13142078 (PMC10384747; doi:10.3390/nano13142078)
Supplement: Supplementary file 1 [file nanomaterials-13-02078-s001.zip › nanomaterials-2470491-supplementary.pdf]

1. SBA-15 MSNs

The XRD patterns, N_2 physisorption isotherms and Raman spectra of the SBA-15 MSNs calcined at 550, 700 and 800 °C are presented in Figures S1, S2 and S3.

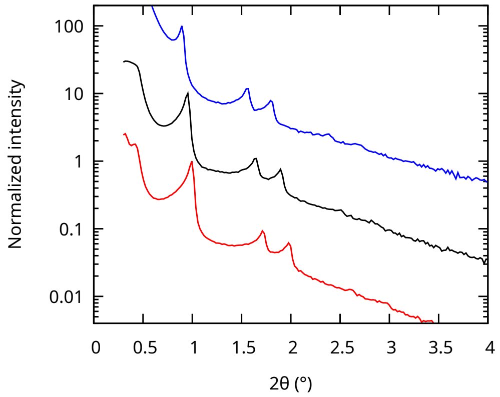

Figure S1. XRD patterns of the SBA-15 MSNs calcined at 800 °C (red), 700 °C (black), 550 °C (blue) from bottom to top. They are vertically shifted for clarity.

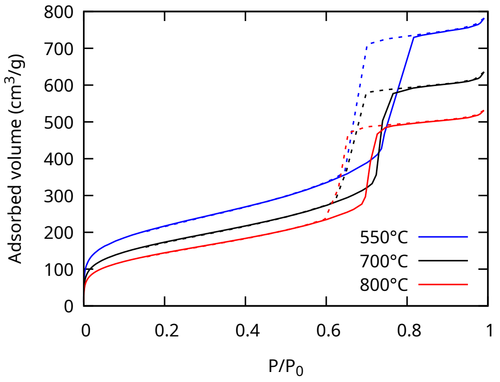

Figure S2. N_2 physisorption isotherms of the SBA-15 MSNs calcined at 800 °C (red), 700 °C (black), 550 °C (blue) from bottom to top. The full and dashed curves are the adsorption and desorption branches of the isotherm respectively.

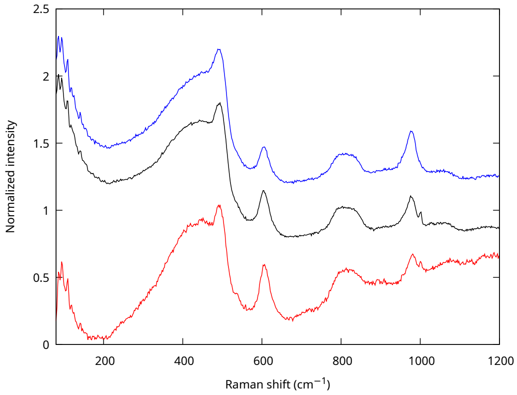

Figure S3. Raman spectra of the SBA-15 MSNs calcined at 550, 700 and 800 °C from top to bottom. The intensities are normalized and the spectra are vertically shifted for clarity.

A representative SEM image of the sample calcined at 700 °C is shown in Figure S4. It shows that the size and shape distributions of the MSNs are quite large. Most of the NPs are elongated with a diameter of about 0.5 µm and lengths of a few µm. The SEM images for the other 2 samples show NPs having similar shapes and dimensions.

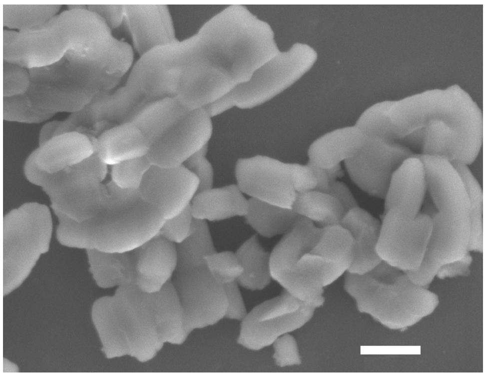

Figure S4. Representative SEM image of the SBA-15 sample calcined at 700 °C (scale bar: 1 µm).

2. SiO_2 NPs

Figure S5 shows the size distribution determined from SEM images and a representa-tive SEM image for the non-porous silica NPs. The NPs are approximately spherical with a narrow size distribution and a 132 nm average diameter.

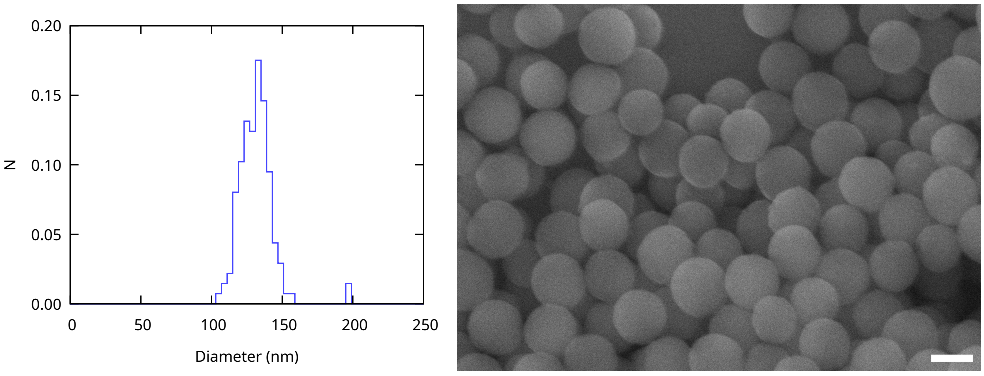

Figure S5. Size distribution and representative SEM image of the SiO_2 NPs (scale bar: 100 nm).

The corresponding Raman spectra are shown in Figure S6. They contain a photoluminescence background and C–H stretching bands near 2900 cm^{-1} due to remaining organic moieties. The SiO_2 network peaks are present. The SiOH band and the D_1 peak are also

present and quite intense showing that the condensation of silica is not as complete as in the previous annealed SBA-15 MSNs. The D_2 peak is not seen.

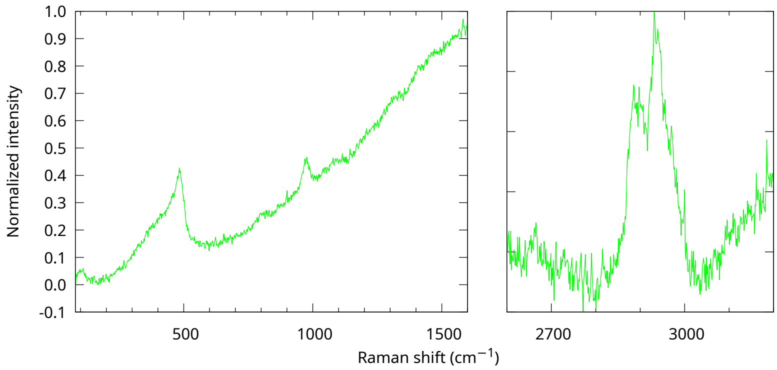

Figure S6. Raman spectra of the SiO_2 NPs (not calcined).

3. Brillouin peaks fitting

To determine the Brillouin peak frequencies, the spectra were fitted using the non-linear least-squares Marquardt-Levenberg algorithm (gnuplot software package version 5.4 patchlevel 4). A lorentzian line shape was used for the Brillouin peak. For the SBA-15 MSNs, the Brillouin and Rayleigh peaks overlap. Therefore, the Rayleigh peak was also fitted with a lorentzian line shape. The results are presented in Figure S7. In all cases, the fit converged and the asymptotic standard error for the peak position was lower than a few %. The frequencies reported in the manuscript correspond to the position of the maxima rounded with 2 significant digits.

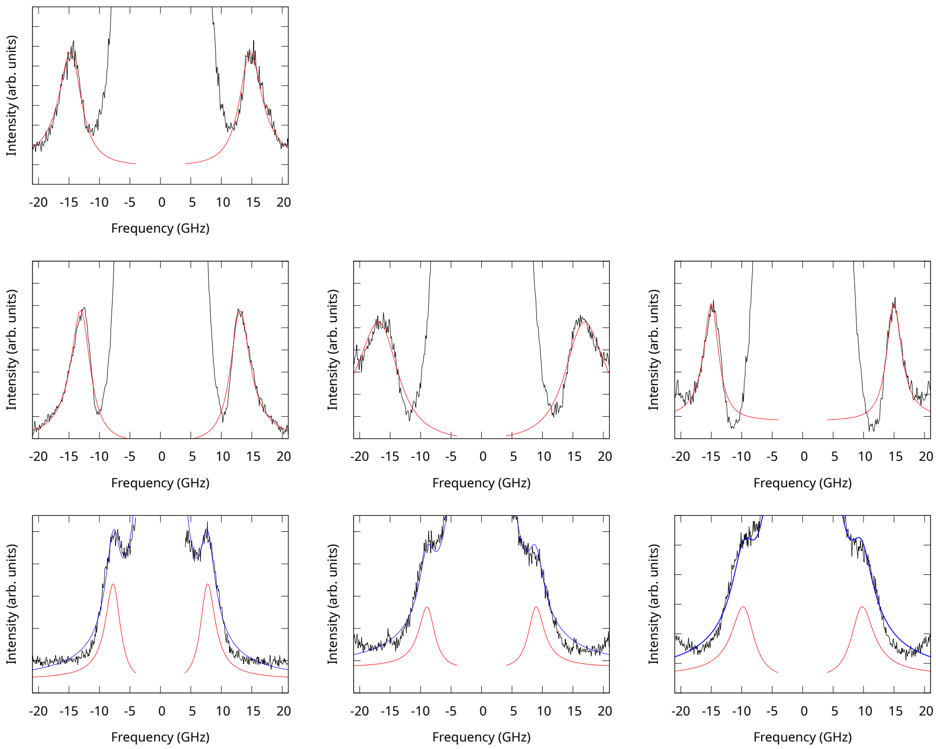

Figure S7. Fit of the Brillouin peaks by a lorentzian shape. The first line is for the non-porous SiO_2 NPs. The second and third lines are for the MCM-41 and SBA-15 MSNs respectively for calcination temperatures 550, 700 and 800 °C from left to right.
